# Are Cockroaches an Important Source of Indoor Endotoxins?

**DOI:** 10.3390/ijerph14010091

**Published:** 2017-01-18

**Authors:** Ka Man Lai

**Affiliations:** Department of Biology, Hong Kong Baptist University, Kowloon Tong, Hong Kong, China; laikaman@hkbu.edu.hk; Tel.: +852-3411-5835

**Keywords:** endotoxins, cockroaches, indoor hygiene, environmental assessment

## Abstract

Endotoxins are common indoor biocontaminants. Their levels have been shown to link to many sources and factors. One of them is cockroach infestation but the role of cockroaches and contamination mechanisms are unclear. We hypothesized that not only is cockroach infestation a sign of poor hygiene, but it also contributes to indoor endotoxins via fecal contamination. In this study, different cockroach species were caught in homes. The endotoxin and allergen levels and their ratios in cockroach feces were determined. To estimate the amount of indoor endotoxins that originated from cockroaches, a new approach of using these new cockroach endotoxin and allergen ratios to compare with environmental data was employed. We found that *Supella (S.) longipalpa*, *Periplaneta (P.) australasiae*, and *Blattella (B.) germanica* were dominant in homes. On average, *P. australasiae* feces had a higher level but greater variation of endotoxins. *B. germanica* feces had the highest levels of allergens measured. Depending on environmental bacterial load and the type of cockroaches present, cockroach endotoxins in the environment may vary greatly. Cockroaches directly contribute to indoor endotoxins rather than just being a sign of poor hygiene. The type and extent of cockroach infestation should be taken into consideration when assessing and remediating indoor endotoxin contamination.

## 1. Introduction

Cockroaches are the most common pests found in human dwellings, as well as many other indoor and outdoor urban environments. The public health significance of cockroaches is mainly focused on their ability to produce potent allergens [1,2] and transmit diseases [3,4]. In this study, it was hypothesized that cockroaches also play a key role in contributing to environmental endotoxins. Endotoxins are lipopolysaccharides naturally present in the outer membrane of Gram-negative bacteria and are ubiquitous indoor biocontaminants that contribute to the development and severity of asthma and other respiratory symptoms [5,6]. Endotoxins are sometimes measured with other allergens in environmental studies to correlate the effect of these hazardous agents and allergens with different health outcomes [7]. In laboratory studies, Kulhankova et al. [8] reported that the co-administration of both cockroach allergens and endotoxins increases pulmonary and systemic responses. Natarajan et al. [9] also demonstrated that endotoxins modulate the allergic reaction to cockroach allergens. 

Several studies have reported that cockroach infestations and stains, along with other factors such as smoking, food debris, cat and dog ownership, dampness, previous water damage, and sampling location in homes, can be used as predictors of endotoxin levels in dust particles [5,10], but no study has traced the role and amount of endotoxins sourced from cockroaches. Is cockroach infestation merely an indicator of poor environmental hygiene or actually a source of endotoxins? Cockroach feces, probably containing allergens and endotoxins from the digestive tract [11], may form respirable particles and enter the human body through inhalation. The aim of this study was to determine endotoxin and allergen levels, and their ratios in cockroach feces. After that, a new approach of using these ratios to compare with environmental data to estimate the cockroach contribution to indoor endotoxins was evaluated. Bla g 1 and Bla g 2 are allergens from *Blattella (B.) germanica* and are both secreted in the digestive tract and excreted with the feces. Other cockroaches may also produce similar allergens that are detectable using the same assays. Therefore, Bla g 1 and Bla g 2 assays were employed in this study, and the measured values were used as cockroach markers. 

## 2. Materials and Methods

### 2.1. Cockroach Sampling

Twenty-two households living in single-family apartments in Hong Kong where the inhabitants had seen cockroaches at home, participated in the cockroach sampling during the summer of 2013. Each household was provided with three sticky traps (Speedtox) to be placed in the kitchen, the bathroom, and other areas where cockroaches had been seen. The sampling time was two days [12]. In total, 66 traps were set up for the study, 43 in the kitchen, 18 in the bathroom, and 5 in the living room. 

### 2.2. Endotoxin and Allergen Extraction and Measurement

Cockroach species were identified and counted according to their morphological features [13]. Feces on traps were picked, weighed (fresh weight), and extracted for allergen and endotoxin analysis following the method of Schram et al. [14]. Endotoxins were extracted with 0.05% Tween-20 in pyrogen-free water, and allergens were extracted with PBS-0.045% Tween-20. Endotoxins and allergens were measured using the kinetic chromogenic Limulus Amebocyte Lysate (LAL) assay (Lonza, Allendale, NJ, USA) and Bla g 1 and Bla g 2 monoclonal antibody-based sandwich ELISA assays (Indoor Biotechnologies, Inc., Charlottesville, VA, USA), respectively. According to the manufacturer, each unit of Bla g 1 measurement is equivalent to about 100 ng of Bla g 1 protein. 

### 2.3. Bacterial Community in the Whole Body Extracts 

*Supella (S.) longipalpa* and *Periplaneta (P.) australasiae* were selected for the whole body metagenomic analysis because of their high abundance in the samples, and the lack of information regarding their microbiota [3,4]. One cockroach body was randomly taken from each trap, and then these bodies were grouped together according to their species. The bodies were crushed in distilled water and the DNA was extracted using a PowerSoil DNA isolation kit following the manufacturer instruction. The 16S rRNA gene was sequenced at the V3–V4 region, using 341 forward and 805 reverse primers on the MiSeq System, following the Illumina Nextera protocol (Illumina, San Diego, CA, USA). Bacterial identification was achieved by matching short subsequences of the reads to a set of 16S reference sequences in the Illumina system Greengenes 13.5 taxonomy database. The number of reads was used to calculate the abundance of the identified bacteria. The details of the 16S metagenomic sequencing library preparation, Illumina metagenomics sequencing procedures, and bioinformatics data analysis were described in Lai et al.’s study [15]. 

## 3. Results 

### 3.1. Endotoxin and Allergen Levels in Feces

Cockroaches were caught in 18 out of 22 selected households (82% sampling rate). The most frequently caught and abundant species were *S. longipalpa* (9 households, 390 organisms, 51% from the living room; 46.7% from the kitchen; 2.3% from the bathroom), and then *B. germanica* (5 households, 89 organisms, 60.7% from the kitchen; 39.3% from the bathroom) and *P. australasiae* (5 households, 76 organisms, 84.2% from the kitchen; 15.8% from the bathroom). The least abundant species was *P. americana* (1 household, 2 organisms, 50% from the kitchen, 50% from the bathroom). Two households had two cockroach species caught at the same time. The combinations of species were *B. germanica* and *P. australasiae*, and *B. germanica* and *S. longipalpa*. Ten traps containing cockroach feces from a single species were selected from six households for further endotoxin and allergen analysis. The sample size was limited because not all cockroaches defecated and not all feces could be gathered successfully. Four *P. australasiae*, three *S. longipalpa*, and three *B. germanica* fecal samples were analyzed and their corresponding endotoxin and allergen levels are shown in Table 1. On average, *P. australasiae* samples had much higher levels (about four times) of endotoxins compared with *S. longipalpa* and *B. germanica*. Moreover, a larger standard deviation was observed in *P. australasiae* samples than in other cockroach samples. The *B. germanica* samples had much higher levels of Bla g 1 and Bla g 2 than *P. australasiae* and *S. longipalpa*.

The ratios between fecal endotoxin, Bla g 1, and Bla g 2 levels were calculated in different cockroach species (Table 1). In general, these ratios had a large standard deviation, except for in *B. germanica* cockroaches. This implies that unknown factors can affect the level of endotoxins and allergens separately. Bla g 2 is specific to *B. germanica*. Although some levels of response were detected in the Bla g 2 assay with *P. australasiae* samples (average = 475 ng·g^−1^), the levels were nearly 65 times lower than in *B. germanica* (30,673 ng·g^−1^).

### 3.2. Bacterial Community in the Whole Body Extracts

All the reads passed through the quality filter and were classified at different taxonomic levels. Table 2 and Table 3 show the top seven bacteria identified in the whole body extracts of *S. longipalpa* and *P. australasiae*, respectively. Less than 25% of the bacterial phyla in the *S. longipalpa* sample were Gram-negative bacteria, while more than 80% of the bacterial phyla in the *P. australasiae* sample were Gram-negative bacteria. Both the Bacteroidetes and Proteobacteria phyla were important in *S. longipalpa*, while over 79% of Gram-negative bacteria came from a single phylum of Proteobacteria in *P. australasiae.* The genus *Pseudomonas* spp. contributed to nearly 60% of the bacteria identified among all other genera in *P. australasiae*.

## 4. Discussion

### 4.1. Cockroaches in Hong Kong and Other Cities Nearby

*S. longipalpa* and *B. germanica* are considered to be the most cosmopolitan species [3]. *P. americana* and *P. australasiae* are present mainly in tropical and subtropical regions (warm and humid conditions), but *P. australasiae* requires warmer temperatures and prefers a diet of vegetation (a more rural species) than *P. americana* [3,16]. *P. americana* is a scavenger, feeding on fermenting food, and is common in urban areas [3,11]. However, this study found more *P. australasiae* than *P. americana* in indoor samples. *P. australasiae* is much less reported and studied worldwide compared to *P. americana*. Comparing the cockroach species between Hong Kong and cities nearby, Tawatsin et al. [17] reported that the dominant species in homes in Thailand were *P. americana* (61%), *P. brunnea* (15%), *Neostypyga rhombiofolia* (9.6%), and *P. australasiae* (9.2%), while *B. germanica* (0.6%) and *S. longipalpa* (0.3%) only accounted for a small percentage. In Kaohsiung, Taiwan, for the cockroaches collected in homes, 54% were *P. americana* and 46% were *B. germanica* [18]. In Penang, Malaysia, the abundant species sampled in homes were *P. americana* (84.3%), *P. brunnea* (9.9%), and *S. longipalpa* (2.4%), while *B. germanica* was not found in the study [16]. The difference observed between the findings in Europe and the USA to that of Asia is that the types of indoor cockroach species are more diverse in tropical and subtropical cities. 

### 4.2. Allergen Levels in Different Cockroach Species 

*B. germanica* samples, compared to those of *P. australasiae* and *S. longipalpa*, were expected to have much higher levels of Bla g 1 and Bla g 2 because the assays were specifically designed for these allergens. It is important to note that the use of Bla g 1 and Bla g 2 assays on *P. australasiae* and *S. longipalpa* feces was not because they produce these allergens. These assays were used to show what the readings would be if other cockroach feces were sampled and tested in field studies. These assays are commonly used in environmental assessments without taking into consideration the type of cockroaches present. Therefore, it is possible that feces from different types of cockroaches were being analyzed in previous studies. 

Group 1 allergens, Bla g 1 and Per a 1 (from *P. americana*) are cross-reactive and share a 70%–72% amino acid sequence identity. Thus, we postulated that other cockroach species may also have similar allergens that can be detected by the Bla g 1 assay [1]. Our results support this postulation. A low but detectable level of reaction, about 15 times less than that in *B. germanica* (564 U·g^−1^), was detected in the *P. australasiae* and *S. longipalpa* samples (32 and 37 U·g^−1^, respectively). Therefore, the use of Bla g 1 in endotoxin calculations for other cockroaches is justified. On the contrary, Bla g 2 shares homology with the aspartic protease enzymes and does not cross-react with other known cockroach allergens [1]. Our results show that *P. australasiae* samples had a lower reading of nearly 50 times in the Bla g 2 assay than the *B. germanica* samples, while the Bla g 2 assay reading was minimal in the *S. longipalpa* samples. These data also explain the previous finding that Bla g 2 is undetectable when cockroach infestation is due to a species other than *B. germanica* [1].

### 4.3. Endotoxin Levels in Different Cockroach Species

*P. australasiae* feces can have a much higher level of endotoxins than that in *S. longipalpa* and *B. germanica* feces. Some studies reported that the bacterial loads in cockroach guts correlate with the bacterial loads in the environment, i.e., cockroaches caught in food handling establishments contain more bacteria in the gut and cuticle than those in human dwellings and hospitals [19,20]. This implies that endotoxin levels in feces may be associated with environmental bacterial load. Previous studies have reported *P. australasiae* to be a rural species, while *S. longipalpa* and *B. germanica* are considered to be indoor species, mostly found in the drier areas of homes and in hotels and restaurants, respectively. Thus, *S. longipalpa* and *B. germanica* potentially carry less environmental bacteria because of these water-limiting conditions [16]. Depending on the environmental conditions, cockroaches consume different levels of bacteria, directly contributing to the endotoxin levels in cockroach feces. Outdoor environments such as sewers and waste deposal sites, where cockroaches may inhabit, are likely to be more diverse in terms of microbial loads compared with indoor environments. This could explain the greater variation of fecal endotoxin levels in *P. australasiae* cockroaches because they live in homes or come from outdoor environments. In our outdoor cockroach studies (data not shown), *P. americana* was the most common species caught followed by *P. australasiae.* Moreover, some bacteria can also be cultivated inside the gut of cockroaches. It is possible that gut physiology and microbial ecosystem contribute to fecal endotoxin levels [21,22,23]. In laboratory-reared *B. germanica* cockroaches, which presumably have been exposed to lower environmental levels of bacteria than the wild ones, the phyla Bacteriodetes (Gram-negative bacteria) and Firmicutes (Gram-positive bacteria) are predominantly contained in the gut, and these microbial compositions change with the life cycle [23]. To further investigate whether the living environment of a cockroach can affect endotoxin levels, we found an interesting study by Tungtrongchitr et al. [11]. They compared endotoxin levels in whole cockroach body extracts of wild- and laboratory-reared American cockroaches. The endotoxin level in the wild cockroaches was 8113 EU·mL^−1^, while the level for those reared in the laboratory was only 863 EU·mL^−1^. This indicates that wild cockroaches contain nearly 10 times more endotoxins in their body than reared cockroaches. In our samples, the lower level of endotoxins in some *P. australasiae* feces might be due to their indoor inhabitation. 

We also determined the bacterial community in the whole body extracts of *P. australasiae* and *S. longipalpa* samples to examine whether the higher endotoxin level in *P. australasiae* correlated with more Gram-negative bacteria and the type of bacteria present. Our results show that the grouped *P. australasiae* sample carries more Gram-negative bacteria (>80%) than the *S. longipalpa* sample (<25%), and so is likely to produce more endotoxin in its feces. These Gram-negative bacteria in *P. australasiae* are mainly *Pseudomonas* spp. (comprising nearly 60% of the identified bacteria among all other genera). *P. fragi* (18.21%), *P. azotoformans* (12.88%), and *P. lundensis* (8.91%) are common bacterial spoilers of food such as dairy products, meats, fish, and rice [24]. This shows that consuming food spoiled by these bacteria may be associated with an increase in endotoxin levels in the feces. Interestingly, this data also demonstrates that *P. australasiae* may consume fermenting food in its diet, as does *P. americana*. In *S. longipalpa*, the major Gram-negative bacterial phylum is Bacteroidetes (12.92%). Proteobacteria (Gram-negative) also contributed to 9.02% of the bacteria in the *S. longipalpa* sample, while the order Enterobacteriales was slightly more abundant than Pseudomonadales, indicating a potential variation in food intake compared with *P. australasiae*. Compared to the gut microbiota of the laboratory-reared *B. germanica* cockroaches, this result implies that *S. longipalpa* cockroaches may inhabit a low bacterial load environment and have a similar diet preference to *B. germanica* cockroaches [23]. 

### 4.4. Environmental Importance of Cockroach Endotoxins 

Environmental endotoxin levels can vary greatly. According to Thorne’s study [5], the weighted geometric mean endotoxin concentration ranged from 18.7 to 80.5 EU/mg among over two thousand house dust samples in the USA. The current study reports that cockroach feces contain at least 950 EU/mg of endotoxins, and the levels can go up to over 30,000 EU/mg. This implies that fecal particles could significantly skew environmental endotoxin levels. When households with heavy cockroach infestation are sampled, the type and extent of cockroach infestation should be taken into consideration when assessing indoor endotoxin contamination. As the feces of *P. australasiae* can contain higher endotoxin concentrations, infestation by this cockroach is likely to increase the cockroach endotoxin levels in the environment. It is plausible that, in tropical and subtropical cities, infestation with cockroaches coming from the outdoor environment can increase the exposure risk to cockroach endotoxins. 

### 4.5. Applying a New Approach to Estimate Cockroach Endotoxins in the Environment 

Finally, we tested the idea that cockroach allergen levels in field samples can be used to estimate the cockroach endotoxin levels in the environment based on our finding that endotoxins and allergens are concurrent in fecal particles. Using a previous environmental study in New Zealand [7] and converting the Bla g 2 level to endotoxins using the ratio of 0.1 EU·mg^−1^ of endotoxin per unit of Bla g 2 (ng·g^−1^) (Table 1), we estimated that 9.67% of the endotoxins in the environment came from *B. germanica* (Table 4). Since the local Public Health Service reports the presence of other cockroach species, it is likely that cockroaches may contribute to an even higher percentage of endotoxins indoors. In the USA and Europe, the most dominant cockroach species sampled in indoor environments was *B. germanica*. As Bla g 2 is specific to *B. germanica,* and this cockroach species was found to be dominant in the environment, the calculation was more straightforward than in the environment with multiple cockroach species. Bla g 1 could be useful in estimating cockroach endotoxin levels if the type of cockroaches present in the environment is known. 

### 4.6. Limitations of Using Bla g 1 and Bla g 2 to Estimate Cockroach Endotoxins 

In this study, only Bla g 2 was used in the estimation of cockroach endotoxin contribution to the environmental samples because studies reported both the types of cockroach infestation and allergen levels are lacking in tropical and subtropical regions. Using Bla g 1 will mean making several assumptions about the type and abundance of cockroaches in the environment. Bla g 2 is commonly measured indoors, but *B. germanica* may not be the dominant species. Several studies in Hong Kong measured Bla g 2 in environmental samples. Leung et al. [26] reported that the kitchen dust contained the highest level of Bla g 2 (mean = 0.2 U·g^−1^, max. = 9.1 U·g^−1^) which, when converted, equates to about 0.02 EU·mg^−1^ of endotoxin from *B. germanica* feces. However, as shown in our findings, other cockroach species may be present in the environment so the contribution of cockroaches to total endotoxin levels in dust cannot be fully determined. Different factors may affect the level of endotoxins and allergens in feces; therefore, using a single endotoxin/allergen ratio may be problematic. For instance, environmental bacterial loads may affect endotoxin levels, while the life cycle and amount of food intake may affect the Bla g 1 level [19,27]. Further investigations are required to study different factors and to refine the application of this approach to assess cockroach endotoxins in the environment. 

## 5. Conclusions

This study demonstrated the importance of cockroach endotoxins in indoor environments. As cockroaches and their endotoxin and allergen contents in feces are likely to vary from place to place, local studies are required to determine the type of cockroaches and their endotoxin and allergen levels in feces so that this information may be used to justify whether cockroach infestation is the main source of endotoxin contamination. 

## Figures and Tables

**Table 1:** Endotoxin and allergen levels in cockroach feces.

Household	Trap Location	Species	Endotoxin (EU·mg^−1^)	Bla g 1 (U·g^−1^)	Bla g 2 (ng·g^−1^)	Endotoxin/Bla g 1 (EU·mg^−1^/U·g^−1^)	Endotoxin/Bla g 2 (EU·mg^−1^/ng·g^−1^)
1	Bathroom	*P. australasiae*	1816	35	692	51.9	2.62
	Kitchen	*P. australasiae*	975	19	200	51.3	4.88
2	Bathroom	*P. australasiae*	21,031	36	568	584	37.0
3	Bathroom	*P. australasiae*	31,292	40	440	782	71.1
	*Average (standard deviation)*		13,779 (14,904)	32.5 (9.26)	475 (210)	367 (373)	28.9 (32.2)
	*Geometric mean (geometric standard deviation)*		5843 (5.67)	31.3 (1.40)	431 (1.73)	187 (4.44)	13.5 (4.86)
4	Bathroom	*S. longipalpa*	3891	53	63	73.4	NA
	Kitchen	*S. longipalpa*	3403	14	< 29	243	NA
	Bathroom	*S. longipalpa*	3241	43	< 29	75.4	NA
	*Average (standard deviation)*		3512 (338)	36.7 (20.3)	NA	131 (97.3)	NA
	*Geometric mean (geometric standard deviation)*		3501 (1.10)	31.7 (2.05)	NA	110 (1.98)	NA
5	Bathroom	*B. germanica*	3040	500	34,500	6.08	0.09
6	Bathroom	*B. germanica*	2993	580	29,834	5.16	0.10
	Bathroom	*B. germanica*	3000	612	27,685	4.90	0.11
	*Average (standard deviation)*		3011 (25.4)	564 (57.7)	30,673 (3484)	5.38 (0.62)	0.10 (0.01)
	*Geometric mean (geometric standard deviation)*		3011 (1.01)	562 (1.11)	30,544 (1.12)	5.36 (1.12)	0.10 (1.11)

NA—Not applicable.

**Table 2:** Bacterial community (Top 7 identifications) in the whole body extract of *S. longipalpa.*

Phylum	Class	Order	Family	Genus	Species
Firmicutes (+ve): 76.05%	Bacilli: 73.40%	Lactobacillales: 72.92%	Enterococcaceae: 46.18%	*Enterococcus*: 44.27%	*gilvus*: 14.12%
					*avium*: 10.92%
			Lactobacillaceae: 25.97%	*Pediococcus*: 14.70%	*cellicola*: 1.70%
					*claussenii*: 1.68%
				*Lactobacillus*: 10.00%	*japonicas*: 3.10%
					*jensenii*: 1.66%
	Clostridia: 2.30%	Clostridiales: 2.25%			
Bacteroidetes (−ve): 12.92%	Bacteroidia: 8.86%	Bacteroidales: 8.86%	Porphyromonadaceae: 6.84%	*Parabacteroides*: 2.68%	
	Flavobacteriia: 3.92%	Flavobacteriales: 3.92%	Blattabacteriaceae: 2.78%	*Blattabacterium*: 2.78%	
Proteobacteria (−ve): 9.02%	Gammaproteobacteria: 6.77%	Enterobacteriales: 3.78%	Enterobacteriaceae: 3.78%	*Enterobacter*: 2.43%	
		Pseudomonadales: 2.68%	Pseudomonadaceae: 2.64%	*Pseudomonas*: 2.64%	*fragi*: 2.01%
	Deltaproteobacteria: 1.58%	Desulfovibrionales: 1.41%	Desulfovibrionaceae: 1.37%		
Verrucomicrobia (−ve): 0.69%	Verrucomicrobiae: 0.68%				
Total identified: 23 *	39	79	172	392	667
Unclassified: 0.63%	0.78%	0.89%	1.81%	4.29%	48.03%

* Others in Phylum: Acidobacteria (−ve): 0.15%, Tenericutes (−ve): 0.14%, and Synergistetes (−ve): 0.12%.

**Table 3:** Bacterial community (Top 7 identifications) in the whole body extract of *P. australasiae.*

Phylum	Class	Order	Family	Genus	Species
Proteobacteria (−ve): 79.63%	Gammaproteobacteria: 78.60%	Pseudomonadales: 60.14%	Pseudomonadaceae: 60.08%	*Pseudomonas*: 59.95%	*fragi*: 18.21%
					*azotoformans*: 12.88%
					*lundensis*: 8.91%
					*moraviensis*: 7.23%
		Enterobacteriales: 16.73%	Enterobacteriaceae: 16.73%	*Enterobacter*: 4.86%	*hormaechei*: 2.70%
				*Citrobacter*: 8.00%	*freudii*: 2.09%
		Aeromonadales: 0.61%	Aeromonadaceae: 0.60%		
	Deltaproteobacteria: 0.46%	Desulfovibrionales: 0.35%			
	Alphaproteobacteria: 0.32%			*Gluconacetobacter*: 0.69%	
Firmicutes (+ve): 16.52%	Bacilli: 16.11%	Lactobacillales: 15.90%	Enterococcaceae: 11.65%	*Enterococcus*: 11.19%	*gilvus*: 8.61%
			Streptococcaceae: 1.99%	*Lactococcus*: 1.96%	
			Lactobacillaceae: 1.95%	*Lactobacillus*: 1.63%	
	Clostridia: 0.31%				
Bacteroidetes (−ve): 2.24%	Bacteroidia: 1.62%	Bacteroidales: 1.62%	Porphyromonadaceae: 1.16%		
	Flavobacteriia: 0.47%	Flavobacteriales: 0.47%			
Total identified: 24 *	40	85	189	455	751
Unclassified: 0.85%	1.04%	1.54%	1.76%	4.76%	26.17%

* Others in Phylum: Fusobacteria (−ve): 0.23%, Actinobacteria (+ve): 0.22%, Nitrospirae (−ve): 0.07%, and Verrucomicrobia (−ve): 0.06%.

**Table 4:** Contribution of cockroach endotoxins to environmental samples.

Cockroaches	Environments Studied	Bla g 1 (U·g^−1^)	Bla g 2 (ng·g^−1^)	Endotoxin (EU·mg^−1^)	Endotoxin from Cockroaches
New Zealand (Auckland Regional Public Health Service) [25]—Common species in homes—*P. americana*, *B. germanica*, *B. asahiniai*, and *Blatta orientalis*	Kindergartens and daycare centres, New Zealand [7]	No data	28	29	9.67% from *B. germanica*. Other cockroach species may be present but the measurement of Bla g 2 alone cannot reveal endotoxin levels from other species.
